# Acute and Long-Term Effects of Aortic Compliance Decrease on Central Hemodynamics: A Modeling Analysis

**DOI:** 10.3389/fphys.2021.701154

**Published:** 2021-07-26

**Authors:** Stamatia Pagoulatou, Dionysios Adamopoulos, Georgios Rovas, Vasiliki Bikia, Nikolaos Stergiopulos

**Affiliations:** ^1^Laboratory of Hemodynamics and Cardiovascular Technology, Institute of Bioengineering, Ecole Polytechnique Fédérale de Lausanne, Lausanne, Switzerland; ^2^Cardiology Department, Geneva University Hospitals, Geneva, Switzerland

**Keywords:** banding, LV remodeling, augmentation index, hypertension, wave separation analysis

## Abstract

Aortic compliance is an important determinant of cardiac afterload and a contributor to cardiovascular morbidity. In the present study, we sought to provide *in silico* insights into the acute as well as long-term effects of aortic compliance decrease on central hemodynamics. To that aim, we used a mathematical model of the cardiovascular system to simulate the hemodynamics (a) of a healthy young adult (baseline), (b) acutely after banding of the proximal aorta, (c) after the heart remodeled itself to match the increased afterload. The simulated pressure and flow waves were used for subsequent wave separation analysis. Aortic banding induced hypertension (SBP 106 mmHg at baseline versus 152 mmHg after banding), which was sustained after left ventricular (LV) remodeling. The main mechanism that drove hypertension was the enhancement of the forward wave, which became even more significant after LV remodeling (forward amplitude 30 mmHg at baseline versus 60 mmHg acutely after banding versus 64 mmHg after remodeling). Accordingly, the forward wave’s contribution to the total pulse pressure increased throughout this process, while the reflection coefficient acutely decreased and then remained roughly constant. Finally, LV remodeling was accompanied by a decrease in augmentation index (AIx 13% acutely after banding versus −3% after remodeling) and a change of the central pressure wave phenotype from the characteristic *Type A* (“old”) to *Type C* (“young”) phenotype. These findings provide valuable insights into the mechanisms of hypertension and provoke us to reconsider our understanding of AIx as a solely arterial parameter.

## Introduction

The proximal aorta is a highly compliant vessel. Due to its elasticity, it can dilate during systole in order to accommodate blood ejected by the heart and thereby dampen the amplitude of the pressure wave (O’Rourke and Hashimoto, 2007). Aortic compliance is, therefore, an important determinant of cardiac afterload.

In healthy young adults, aortic compliance accounts for more than half of the arterial system’s total compliance (Ioannou et al., 2003). Nevertheless, several processes, pathologies, and surgical interventions can significantly reduce aortic elasticity (Boutouyrie et al., 1992; Kimoto et al., 2003; Vardoulis et al., 2011), leading to increased cardiovascular risk (Vlachopoulos et al., 2010; Cecelja and Chowienczyk, 2012). One such surgical intervention is aortic reconstruction with non-compliant prosthetic grafts, typically performed in patients with aortic aneurysm (Etz et al., 2007). Following proximal aortic bypass procedures, hypertension and left ventricular (LV) hypertrophy are often developed in such patients due to the substitution of the compliant native tissue with a stiff graft (Morita et al., 2002; Spadaccio et al., 2016).

**FIGURE 1:**
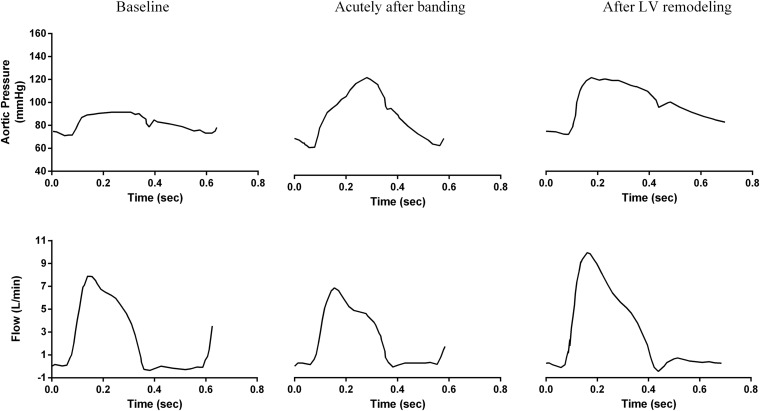
Acute and long-term changes of central aortic pressure and flow due to aortic banding as measured invasively by Ioannou et al. (2009) in the swine. Figure reproduced with permission.

In a previous work, Ioannou et al. (2003, 2009) performed invasive measurements of proximal aortic flow and pressure waves in the swine in order to characterize hemodynamic changes following proximal aortic banding. Aortic banding was implemented to stiffen the proximal aorta in a non-stenotic fashion. Compliance was reduced by 49 ± 9%. Ioannou et al. (2003, 2009) demonstrated that, as expected, pulse pressure increased immediately after the banding procedure, and the aortic pressure wave shape was transformed to the characteristic “old-age” phenotype with a pronounced late systole pressure peak (Ioannou et al., 2003, 2009; Figure 1). They also documented LV hypertrophy signs in the long-term, while the banding-induced systolic hypertension, which sustained for at least 60 days post-operatively. Interestingly, this did not hold for the aortic pressure wave shape, which returned to its original “young-age” phenotype (Ioannou et al., 2009; Figure 1).

Extending on this previous study, the present work aimed to provide *in silico* insights into the acute as well as long-term effects of aortic compliance decrease by leveraging a mathematical model of the cardiovascular system and standard wave separation analysis. The specific goals were to (1) simulate the hemodynamics immediately after banding as well as after LV remodeling due to pressure overload, (2) explain the acute and chronic mechanisms of hypertension following aortic banding, and (3) explain the observed change in the aortic pressure wave phenotype after LV remodeling.

## Materials and Methods

### Brief Description of the Mathematical Model of the Cardiovascular System

The mathematical model used in the present work is based on previous work conducted in our laboratory (Reymond et al., 2009, 2011). This model of the cardiovascular system includes a detailed description of the circulation in the main systemic arteries as well as a model for cardiac contractility. Blood circulation in the arterial network is described by the one-dimensional (1-D) form of the Navier–Stokes equations coupled with a constitutive law for the arterial wall elasticity. At the terminal sites, the arteries are connected with three-element Windkessel models that represent the periphery. This arterial model has been thoroughly validated against *in vivo* human data and has been demonstrated to accurately predict pressure and flow curves throughout the arterial network (Reymond et al., 2009, 2011).

At the proximal boundary, the arterial tree is connected to a 0-D model of the heart’s LV, which is represented by a time-varying elastance function (Suga and Sagawa, 1974; Sagawa et al., 1977). A pressure source feeds the cardiac model, assumed to have a constant value (filling pressure – Pfill). The systolic function of the LV is dictated by a linear end-systolic pressure-volume relation (*ESPVR*) equal to *E*_*es*_⋅(*V*_*LV*_−*V*_*d*_), where *E*_*es*_ is the end-systolic elastance and *V_d* is the dead volume (Sagawa et al., 1977). The diastolic relaxation is described by an exponential end-diastolic pressure-volume relation (EDPVR) equal to *P*_0_⋅exp(β⋅*V*_*LV*_), where *P_0* is the dead pressure and β a diastolic stiffness parameter (Burkhoff et al., 2005). At any given moment, the pressure-volume relation is described by the combination of the ESPVR and EDPVR, weighted according to a time-varying activation function, ∈ (*t*):

$$P_{L\,V}\left(V_{L\,V}\right)=\in \left(t\right)\cdot ESPVR+\left(1-\in \left(t\right)\right)\cdot EDPV$$

### Simulation Setup

#### Baseline

The baseline simulation was set up to represent a healthy young person (Table 1). More specifically, the total peripheral resistance was set at 0.92 mmHg s/mL, and the total arterial compliance was 0.96 mL/mmHg. The cardiac properties were chosen based on previous literature’s physiological ranges (Senzaki et al., 1996; Chen et al., 2001). The systolic function was defined by an end-systolic elastance of 3.2 mmHg/mL and a dead volume of 15 mL. The EDPVR was defined by *P*_0_ 2.3 mmHg and β = 0.013 mL^–1^ (Kadry et al., 2020), while the Pfill was set at 11.5 mmHg (Brinke et al., 2010). This resulted in a stroke volume (SV) of 74 mL and an ejection fraction (EF) of 61% (Lang et al., 2015).

**TABLE 1:** Simulation parameters for baseline, acutely after banding, and after LV remodeling.

Parameter	Baseline	Banding	LV remodeling
Ees (mmHg/mL)	3.2	3.2	4.3
Vd (mL)	15	10	10
Pfill (mmHg)	11.5	12.5	16.5
Po (mmHg)	2.3	2.3	2.3
β (mL^–1^)	0.013	0.013	0.018
EDV (mL)	123	130	118
EF (%)	61%	57%	62%
SV (mL)	74	74	74
CT (mL/mmHg)	0.96	0.58	0.58
TPR (mmHg s/mL)	0.92	1.20	1.20

*Ees, end-systolic elastance; Vd, dead volume; Pfill, filling pressure; Po, dead pressure of EDPVR; b, stiffness parameter of EDPVR; EDV, end-diastolic volume; EF, ejection fraction; SV, stroke volume; CT, total arterial compliance; TPR, total peripheral resistance.*

#### Aortic Banding

**FIGURE 2:**
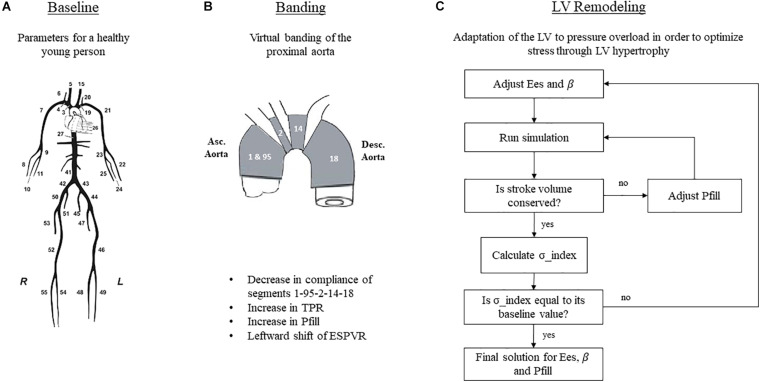
Simulation setup for the three hemodynamic states. **(A)** Schematic representation of the arterial tree, taken from Reymond et al. (2009) and reproduced with permission. **(B)** Simulation of aortic banding. **(C)** Simulation of LV remodeling. TPR, total peripheral resistance; Pfill, filling pressure; Ees, end-systolic elastance; β, diastolic stiffness.

Aortic banding was induced by changing the compliance of only the proximal part of the aorta, composed of the segments 1 – 95 – 2 – 14 – 18 of the arterial tree as described by Reymond et al. (2009; Figure 2). The extent to which these aortic segments were stiffened was chosen based on previous literature (Ioannou et al., 2003). More specifically, the publication of Ioannou et al. (2003) showed *in vivo* that aortic banding induced a decrease in total arterial compliance of approximately 40%. In order to achieve this in our simulation, we had to reduce the compliance of the aortic root by 80%, a value that agrees well with previous publications (Ioannou et al., 2003, 2009; Vardoulis et al., 2011). Total arterial compliance, therefore, decreased from its baseline value of 0.96 to 0.58 mmHg/mL after banding (Table 1).

With respect to the total peripheral resistance, a previous work (Ioannou et al., 2003) showed an increase in the mean arterial pressure (MAP) after banding by 30%, while the cardiac output remained essentially unchanged. In order to capture this in our simulation, we augmented the total peripheral resistance by 30% (Table 1).

**FIGURE 3:**
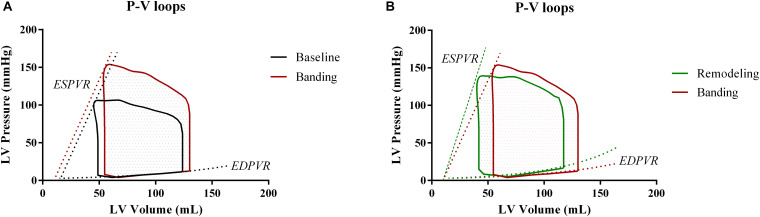
Comparison of the pressure-volume loops at the three different phases. **(A)** Baseline versus acutely after banding. **(B)** Acutely after banding versus after LV remodeling under the form of concentric hypertrophy.

Acutely after banding, the afterload is increased; hence, the heart is operating under higher pressures. Previous publications (Sunagawa et al., 1985; Freeman, 1990) have shown that the LV performance appears enhanced after acute afterload augmentation; this is achieved by a leftward shift of the ESPVR in the P-V plane, without however a concurrent change in its slope. We incorporated this effect in our simulation by decreasing the dead volume, Vd, by 5 mL while maintaining the Ees unchanged in accordance with the findings of Freeman (1990). Given this new ESPVR, the LV needs to increase its end-diastolic volume and pressure in order to maintain the cardiac output, as dictated by the Frank-Starling mechanism (Figure 3). To achieve this in our simulation, we fine-tuned the Pfill parameter, Pfill, so that the generated SV would be conserved, i.e., Pfill was increased from 11.5 to 12.5 mmHg. This resulted in an augmentation of the EDV from 123 to 130 mL and reduced the EF from 61 to 57%.

#### Remodeling

Previous literature has shown that acute changes in the aortic compliance activate mechanisms that strive to restore the matching between the heart and the vascular system (London, 1998). In the case of pressure overload, the optimization of the cardiovascular function is achieved by structural changes, i.e., remodeling of the LV, typically in the form of concentric hypertrophy (Devereux et al., 1987; Ioannou et al., 2009). This compensatory mechanism aims at maintaining the tensile stress acting on the ventricular wall within the normal range, as explained by the law of Laplace. The law of Laplace dictates that tensile stress be directly proportional to pressure and radius and inversely proportional to wall thickness. Therefore, in response to increases in pressure, the ventricular wall needs to thicken in order for the tensile stress to be maintained constant.

In the present study, we simulated the long-term effects of banding following the afore-described paradigm. Concretely, we hypothesized that the hypertrophic heart would also be stiffer, i.e., will have an increased LV end-systolic elastance. The E_*es*_ increase was assumed proportional to the increase in wall thickness (Ganau et al., 1990; Pagoulatou and Stergiopulos, 2017). Furthermore, we hypothesized that ventricular stiffening would also impair the diastolic function of the heart, whereby the stiffness parameter β will increase proportionally to E_*es*_ (Røe et al., 2017). Of note, during remodeling, the SV produced by the heart is conserved, as highlighted in the publication of Ioannou et al. (2009).

Figure 2C explains the iterative scheme that was followed in order to implement the remodeling process. First, we defined a quantity, hereby called stress index, as σ_index = P⋅(EDV)^1/3^/E_*es*_. This equation is equivalent to the formula of Laplace, assuming that the LV radius is proportional to (EDV)^1/3^ and the wall thickness is proportional to E_*es*_. The stress index was calculated at baseline, and this value was set as the remodeling target. Subsequently, we introduced banding and initiated the remodeling stress optimization loop. In this loop, E_*es*_ and β were first arbitrarily increased to values Ees_*remodel*_ and β_*remodel*,_ and the simulation ran. The model predicted a SV that was not equal to its baseline value, given that the stiffer heart needs to increase its end-diastolic pressure in order to achieve the same perfusion. To correct for this, the Pfill was tuned in an internal optimization loop. After the internal optimization converged, the simulation yielded the correct SV, and the aortic pressure was exported. The new stress index was calculated as P_*remod*_⋅(EDV)^1/3^_*remod*_/Ees_*remod*_ and was compared to the baseline value. If the error was higher than 1%, E_*es*_ and β were updated, and a new optimization cycle was initiated. After a few iterations, the scheme converged to a solution set for E_*es*_, β, and Pfill. Note that throughout this manipulation, the dead volume, as well as the parameters of the arterial tree, were kept constant.

The final simulation parameters are summarized in Table 1. Additionally, Figure 3 depicts the simulated pressure-volume loops for the three hemodynamic states.

### Analysis of Hemodynamics

The simulation results for the three hemodynamic states were processed using pulse wave analysis. Concretely, we calculated the following hemodynamic parameters: (1) aortic systolic blood pressure (aSBP), (2) aortic diastolic blood pressure (aDBP), (3) MAP, (4) aortic pulse pressure (aPP), 5) augmentation pressure (AP) defined according to the characteristic inflection point or “shoulder” on the aortic pressure waveform as proposed by Murgo et al. (1980), (6) augmentation index (AIx) calculated as the ratio of the AP over the pulse pressure (Murgo et al., 1980). Additionally, the pressure waveforms were classified to either *Type A* or *Type C* based on the timing of the inflection, where the *Type A* phenotype has the peak systolic pressure occurring after the shoulder and *cAIx*10%, and the *Type C* pressure waveform has the peak systolic pressure preceding the inflection point and *cAIx*0 (Murgo et al., 1980).

The aortic pressure and flow waves were also used in a subsequent frequency-based wave separation analysis. The input impedance was calculated as the ratio of the Fourier transformed pressure over flow signals. The characteristic impedance (Zc) was isolated after averaging the input impedance modulus in the frequency range from 3 to 9 harmonics (Westerhof et al., 1972). The total pressure wave was then separated into its forward and backward components as proposed by Westerhof et al. (1972):

$$P_{f\,o\,r\,w\,a\,r\,d}=\frac{P+Z_{c}\,Q}{2}\,a\,n\,d\,P_{b\,a\,c\,k\,w\,a\,r\,d}=\frac{P-Z_{c}\,Q}{2}$$

The backward and forward wave amplitudes were computed as well as their ratio, hereby called the reflection coefficient. The forward wave’s relative contribution to the total pulse pressure was also calculated as the ratio of the forward wave amplitude over the pulse pressure, PPf/PP.

### Sensitivity Analysis

The complete analysis as described in Figure 2 was repeated for two additional baseline model configurations. More specifically, the short-term and long-term effects of an 80% decrease in proximal aortic compliance were investigated *de novo* using an average 30 and 70 year-old model, according to our previously published aging cardiovascular model (Pagoulatou and Stergiopulos, 2017). The arterial parameters of these models were adjusted according to previous literature, i.e., arterial compliance was adjusted based on the expected evolution of central and peripheral pulse wave velocity with age and peripheral resistance was tuned to achieve the expected increase in MAP. Venous return was increased with aging in order to keep the cardiac output constant despite the increased afterload. Systolic and diastolic LV properties were also altered to incorporate the effects of age-induced hypertrophy and diastolic stiffening. More details on the derivation of the aging models can be found in the original publication (Pagoulatou and Stergiopulos, 2017).

## Results

**FIGURE 4:**
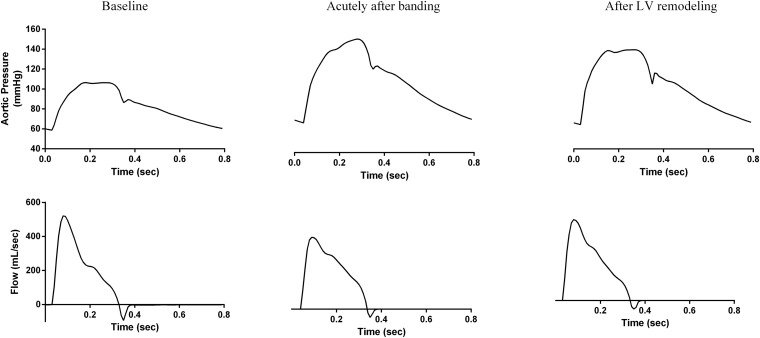
The simulation-generated aortic pressure and flow waves for the three hemodynamic states. **(left)** Baseline, (**center**) acutely after banding, and **(right)** after LV remodeling.

The key hemodynamic characteristics of the three simulations are summarized in Table 2, and the respective aortic pressure and flow curves are shown in Figure 4.

**TABLE 2:** Hemodynamic characteristics at baseline, acutely after banding, and after LV remodeling.

Parameter	Baseline	Acutely after banding	After LV remodeling
Aortic SBP (mmHg)	106	152	140
Aortic DBP (mmHg)	59	68	65
MAP (mmHg)	83	110	104
Aortic PP (mmHg)	48	84	75
Timing of peak pressure (s)	0.18	0.26	0.16
Pressure at inflection (mmHg)	105	141	138
Timing of inflection point (s)	0.21	0.17	0.19
Augmentation Pressure (mmHg)	-1	11	-2
AIx (%)	-2.1	13.1	-2.7
Zc (mmHg s/mL)	0.05	0.16	0.16
Max flow (mL/s)	521	412	476

*SBP, systolic blood pressure; DBP, diastolic blood pressure; MAP, mean arterial pressure; PP, pulse pressure; Aix, augmentation index; Zc, characteristic impedance.*

As expected, the decrease in arterial compliance due to aortic banding induced hypertension and caused significant changes to the pressure wave morphology both acutely and in the long-term. Immediately following banding, aortic SBP and PP increased from 106 to 152 mmHg and from 48 to 84 mmHg, respectively. Concurrently, MAP increased from 83 to 110 mmHg. Hypertension was sustained after LV remodeling, although these pressure changes were slightly mitigated, i.e., aortic SBP dropped to 140 mmHg, PP to 75 mmHg, and MAP to 104 mmHg. The aortic DBP was only slightly affected throughout the process; it increased from its baseline value of 59 to 68 mmHg acutely after banding and remained practically constant after LV remodeling. These pressure alterations were linked with a significant increase in the aortic Zc, which is inversely related to aortic compliance; Zc increased from 0.05 to 0.16 mmHg s/mL acutely after banding and remained constant thereafter.

We observe a very close qualitative agreement between the simulation-predicted waveforms and the experimental findings of Ioannou et al. (2009; Figures 1, 4). As shown in Figure 4 and Table 2, the aortic pressure wave at baseline had a pronounced upstroke with an early peak at 0.18 s and a negative AIx of −2.1%, i.e., the characteristic *Type C* phenotype. Immediately following banding, the pressure wave was characterized by a late systolic peak occurring at 0.26 s and a high positive AIx value of 13.1%, which are indicative of the *Type A* phenotype. Interestingly, after LV remodeling, the simulation predicted the same pressure evolution as during the experiment (Ioannou et al., 2009; Figures 1, 4): the pressure waveform was restored to its original shape (*Type C*), even though the arterial properties were not changed. Similarly, we noted a decrease of the peak flow immediately following banding, which was restored after LV remodeling (Figures 1, 4).

**FIGURE 5:**
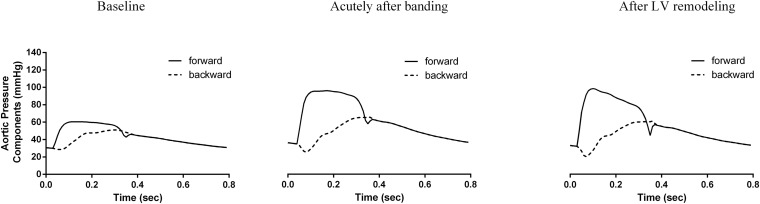
Analysis of the pressure wave into its forward (continuous line) and backward component (dashed line) for the three generated hemodynamic states.

Figure 5 and Table 3 compare the calculated forward and backward pressure wave components between the pre- and post-banding states. Banding leads to a significant increase in the amplitude of both the forward and backward pressure wave components. The forward wave amplitude increased from 30 to 60 mmHg immediately following banding and to 64 mmHg after LV remodeling. The backward wave amplitude rose from 22 to 41 mmHg following banding and further to 42 mmHg. However, the amplitudes ratio, which served as a simplified reflection coefficient, decreased only minimally acutely after banding and then remained roughly constant after LV remodeling.

**TABLE 3:** Features of the forward and backward pressure wave components.

Parameter	Baseline	Acutely after banding	After LV remodeling
Forward wave amplitude (mmHg)	30	60	64
Backward wave amplitude (mmHg)	22	41	43
Forward wave peak (mmHg)	60	94	99
Backward wave peak (mmHg)	51	65	62
Timing of the peak of the forward wave (s)	0.11	0.12	0.10
Max slope of the forward wave (mmHg/s)	0.8e3	1.5e3	2.2e3
Reflection coefficient	0.73	0.67	0.68
Forward wave amplitude/PP	0.63	0.71	0.85

Interestingly, as we can observe in Table 3, the forward wave’s relative contribution to the total pulse pressure increased throughout this process, from 0.63 at baseline to 0.71 after banding and further to 0.85 after LV remodeling. Additionally, the forward wave shape was also altered between the acute and long-term stages; after cardiac adaptation, it had a higher peak value (99 mmHg after remodeling versus 94 mmHg immediately post-banding). Furthermore, the forward pressure wave had its peak occur earlier after remodeling (0.10 versus 0.12 s immediately post-banding), and its upstroke became significantly steeper (2.2 × 10^3^ versus 1.5 × 10^3^mmHg/s, immediately post-banding).

### Sensitivity Analysis

Table 4 summarizes the simulation results we obtained after using the model configurations for a 30 and 70-year old adult for (i) baseline, (ii) acutely after banding, and (iii) after LV remodeling. Acutely after banding, we note for both cases a significant decrease in the total arterial compliance accompanied by an increase in the Zc, which is however more prominent for the young compliant model (loss of 46% of the total arterial compliance for the young subject versus 30% for the old subject). Aortic banding leads to an acute increase in the AP and AIx, which is subsequently alleviated after LV remodeling for both models, although the effect is less visible for the old model (Table 4). Additionally, both cases display the expected trend for an increase in SBP and PP with banding, which is driven by the significant enhancement of the forward pressure wave. Indeed, the contribution of the forward wave to the total pulse pressure increases throughout the manipulation for both cases, for the young subject from 0.69 at baseline to 0.78 acutely to 0.88 after LV remodeling and for the old subject from 0.74 at baseline to 0.77 acutely to 0.82 after LV remodeling.

**TABLE 4:** Simulation results for a young and old adult for baseline, acutely after banding and after LV remodeling.

	Average 30-year old			Average 70-year old		
Parameter	Baseline	Acutely after banding	After LV remodeling	Baseline	Acutely after banding	After LV remodeling
**Modeling parameters**						
Ees (mmHg/mL)	2.5	2.5	3.2	3.0	3.0	3.8
Vd (mL)	15	10	10	15	10	10
Pfill (mmHg)	11.5	12.5	16	13	13.7	19.5
Po (mmHg)	2.3	2.3	2.3	2.3	2.3	2.3
β (mL^–1^)	0.013	0.013	0.017	0.015	0.015	0.018
EDV (mL)	124	130	118	122	125	118
EF (%)	55	52	57	55	53	56
SV (mL)	68	68	68	66	66	66
CT (mL/mmHg)	1.6	0.86	0.86	0.90	0.62	0.62
TPR (mmHg s/mL)	1.0	1.3	1.3	1.22	1.47	1.47
**Aortic flow and pressure**						
Aortic SBP (mmHg)	99	130	121	123	154	150
Aortic DBP (mmHg)	70	81	78	74	81	78
MAP (mmHg)	81	106	101	98	105	102
Aortic PP (mmHg)	29	49	43	49	73	72
Timing of peak pressure (s)	0.18	0.23	0.17	0.23	0.25	0.24
Pressure at inflection (mmHg)	97	114	117	115	141	141
Timing of inflection point (s)	0.14	0.08	0.12	0.14	0.13	0.16
Augmentation pressure (mmHg)	2	16	3	7	15	9
AIx (%)	6.9	32.6	9.5	14.6	20.5	12.5
Zc (mmHg s/mL)	0.04	0.12	0.12	0.11	0.18	0.18
Max flow (mL/s)	408	335	380	390	327	381
**Wave separation**						
Forward wave amplitude (mmHg)	20	37	38	37	56	59
Backward wave amplitude (mmHg)	14	23	25	24	37	38
Forward wave peak (mmHg)	55	78	76	73	96	98
Backward wave peak (mmHg)	46	58	56	56	66	67
Timing of the peak of the forward wave (s)	0.10	0.16	0.10	0.10	0.16	0.10
Max slope of the forward wave (mmHg/s)	5.3e2	9.5e2	11.0e2	1.1e3	1.4e3	2.6e3
Reflection coefficient	0.70	0.62	0.66	0.67	0.60	0.64
Forward wave amplitude/PP	0.69	0.78	0.88	0.74	0.77	0.82

## Discussion

### Main Findings

In this work, we investigated the acute and long-term impact of proximal aortic compliance decrease on central arterial hemodynamics. Concretely, we used a physiologically relevant mathematical model of the cardiovascular system to simulate compliance decrease due to non-stenotic aortic banding and the subsequent remodeling of the LV as a response to pressure overload. We demonstrated that our mathematical model’s predictions were highly consistent with aortic banding experiments in swine. The major findings of this study can be summarized as follows: (a) reduction of the proximal aortic compliance leads acutely to hypertension, with an increase in the aortic SBP and PP, which is sustained yet slightly alleviated after LV remodeling, (b) the primary mechanism for the increase in PP due to banding is an increase in the forward wave amplitude, which is even more enhanced after LV remodeling, (c) additionally to an increase in its magnitude, the forward pressure wave alters its shape after LV remodeling, adopting a pronounced upstroke and an earlier peak, and (d) LV remodeling is the principal cause of the transformation of the pressure waveform from the *Type A* to *Type C* phenotype.

This study supplements previous literature that has explored acute and chronic effects of reduced aortic compliance on hemodynamics by making use of the banding procedure. In the present, aortic banding was achieved via an 80% reduction of the proximal aortic compliance, which yielded a 40% reduction in the total arterial compliance. This is in line with the current thinking that the proximal aorta accounts for approximately half of the total arterial compliance.

Note that in this work, only proximal aortic banding was considered while peripheral compliance was left unaltered. The motivation behind this choice comes from the facts that (a) the compliance of the proximal aorta is a major component of afterload regulating pulsatility, and (b) there are processes, such as physiological aging or aortic reconstruction with prosthetic grafts, which decrease mainly or even exclusively the proximal aortic compliance, thereby impairing cardiovascular function.

### Mechanisms of Hypertension

As expected, aortic compliance reduction was immediately linked with an increase in systolic blood pressure and pulse pressure. This can be understood as the result of an increase in the aortic Zc and the subsequent amplification of the forward pressure wave (Figure 5). Indeed, in our analysis, the forward wave amplitude was almost doubled after banding and the relative contribution of the forward wave to the total pulse pressure increased by 13%. The amplified forward wave also drove the amplification of the backward wave. However, there was a minor change in the relative ratio between the two wave amplitudes, i.e., the reflection coefficient. We might attribute this minor effect on the value of the reflection coefficient to the fact that the major reflection sites in the arterial circulation are at the level of the abdominal bifurcations and other peripheral sites, the geometry and compliance of which was not altered in our simulations.

Our findings have important implications for our understanding of the development of hypertension, particularly for the paradigm of cardiovascular aging. Concretely, the aging process is associated with progressive stiffening of the arteries (14) and the subsequent gradual increase in the central SBP and PP with advancing age, often referred to as isolated systolic or “old age” hypertension (Yip et al., 2009). Traditionally, it is hypothesized that the stiffer arterial tree produces pronounced reflections (O’Rourke and Nichols, 2004). Hypertension is, therefore, the result of the arrival of these augmented reflections back to the heart. Nevertheless, in this line of thinking, we do not take into consideration the fact that aging induces non-uniform arterial stiffening, whereby the proximal aorta becomes significantly stiffer and peripheral compliance hardly changes (Boutouyrie et al., 1992; Kimoto et al., 2003). In light of the evidence provided in the present study, it is likely that old age hypertension might be primarily the result of an augmentation of the forward wave ejected by the heart due to proximal stiffening rather than solely increased reflections coming back from the periphery. This hypothesis has also been investigated and proven plausible in our previous modeling work (Pagoulatou and Stergiopulos, 2017). Similar findings of the relative contribution of the forward wave to the development of hypertension have also been reported in clinical studies on old adults (Mitchell et al., 2004) as well as children and adolescents (Zocalo et al., 2018).

### LV Remodeling and the Forward Wave

The heart and the arterial network form a coupled system and adapt to maintain optimal coupling conditions. Increased arterial stiffness and hypertension can trigger LV remodeling under the form of concentric hypertrophy, which is a powerful mechanism to normalize tensile stress exerted on the heart. This mechanism has been thoroughly described in the literature; it is supported by multiple previous observations, including evidence of increased wall thickness under pressure overload, and at later stages LV dilatation (Xie et al., 2013). In the present work, we simulated LV adaptation in response to arterial compliance decrease and the resulting pressure increase, similarly to our previous computational efforts (Pagoulatou and Stergiopulos, 2017). Our algorithm predicted that a compliance decrease by 40% leads to systolic pressure increase by 42%; the LV needs to increase its wall thickness and, consequently, its contractility by 35% to normalize tensile stress. This result is consistent with prior evidence highlighting the interaction between hemodynamic load and myocardial contractile state (Burkhoff et al., 2005).

Our analysis showed that the primary mechanism that drives the increase in SP and PP acutely after banding is the enhancement of the forward wave amplitude. After LV remodeling, there is an even more pronounced increase in the forward pulse pressure while the total pulse pressure decreases slightly. In other words, the forward wave accounts for a larger part of the total pulse pressure once the heart becomes stiffer. This finding is supported by our previous numerical and clinical observations (Pagoulatou et al., 2020), demonstrating that increased cardiac contractility leads to the generation of a more pronounced forward traveling wave, which is both larger in amplitude and steeper, both of which are direct consequences of the increase in LV contractility.

Building on this framework, we can also offer a plausible explanation as to why the pressure wave phenotype is altered after LV remodeling. The blood pressure and flow at each time point result from the interaction between the heart and the arterial system. At baseline, these two systems operate under optimal coupling conditions. When aortic banding is performed, the aortic compliance instantaneously decreases to a great extent. This entails that the coupling conditions between the heart and the arterial tree will change and likely become less favorable. Indeed, acutely after performing aortic banding, the aortic pressure phenotype has the characteristics of those found in patients with isolated systolic hypertension (Murgo et al., 1980) with a pronounced late systolic peak, i.e., the *Type A* phenotype. Subsequently, the pressure overload will trigger LV remodeling aiming to restore the matching between the two systems. When the heart becomes stiffer and more contractile, it will be able to meet the increased afterload by pumping a steeper, more pronounced forward pressure wave. It is precisely this alteration in the shape of the generated forward wave that drastically alters central hemodynamics, restoring the *Type C* pressure phenotype. These theoretical findings match very well the results obtained in the swine aortic banding experiments. We also reported similar acute changes in the pressure phenotypes and the forward wave amplitude on aortic valve stenosis patients undergoing transcatheter aortic valve replacement (TAVR) (Pagoulatou et al., 2020).

In light of this evidence, we might need to reconsider our understanding of *Type A* and *Type C* pressure phenotypes. Indeed, pressure waveforms characterized as *Type A* are often linked to old individuals with stiff arteries and increased wave reflections. At the same time, the *Type C* phenotype is assumed representative of young adults with elastic arteries and small reflections (Murgo et al., 1980). Here, we demonstrate that these phenotypes are, in fact, the result of the coupling between the cardiac and arterial systems and therefore depend on both components. The same comment can be extended for the AP and AIx, which are traditionally considered sole arterial measures dependent on reflections (Vlachopoulos et al., 2011).

### Sensitivity to Modeling Parameters: Aging Effects

The main study conclusions regarding the acute and long-term effects of afterload increase on central hemodynamics were confirmed after repeating the analysis for different baseline models (Table 4). More specifically, after using the parameters for the average 30-year old and 70-year old subject, we were able to verify that hypertension induced after aortic banding is achieved by an increase in the forward wave amplitude, which becomes the major contributor to total PP after LV remodeling. Interestingly, this mechanism seems to attenuate with advancing age (Table 4). This may be attributed to the fact that the proximal aorta of a young person is highly compliant and therefore aortic banding is expected to increase disproportionally the aortic impedance as compared to an older subject. Additionally, for all hemodynamic cases, we found that the shape of the pressure wave is similarly affected by the afterload increase and the subsequent adaptation of the LV, i.e., the AIx is increased acutely after banding and thereafter restored due to LV remodeling. Interestingly, the later observation is less prominent for the old subject, whose pressure waveform belongs to the characteristic *Type A* phenotype already at baseline.

### Limitations

When interpreting our results, the reader should consider that the data presented above reflect computational simulations. Nevertheless, this limitation is mitigated by the following facts: (a) the state-of-the-art model used here has been thoroughly validated and found capable of accurately representing the hemodynamics of healthy young as well as old individuals (Reymond et al., 2011; Pagoulatou and Stergiopulos, 2017), (b) the simulations were set up in order to closely imitate plausible hemodynamic states, based on the reported literature and widely accepted remodeling mechanisms, (c) the simulation results were in perfect qualitative agreement with previous experimental data performed in swine (Ioannou et al., 2009).

In our simulations, only proximal aortic stiffening was imposed while peripheral circulation was left unaltered. However, it is known that the peripheral compliance and geometry might adjust to increases in afterload, which could have important implications on the wave reflection profile. Moreover, previous studies have shown a marginally significant increase in the heart rate due to the banding of the aorta (Ioannou et al., 2009). This constitutes an acute mechanism of adaptation to the increased afterload, and its effect wears off in the long-term (Ioannou et al., 2009). As we had no available data that would allow us to simulate this compensatory mechanism, we decided to exclude it altogether from our study.

Previous literature has shown that vascular smooth muscle activation during hypertension can acutely improve wall buffering function by attenuating pressure oscillations and diminishing the stress tension on the vascular wall (Grignola et al., 2007). Additional long-term compensatory mechanisms that are related to the nervous system pressure regulation might come into play and alter cardiac contractility, arterial properties, preload and cardiac output (Nobrega et al., 2014). These mechanisms are not included in our computational model, which constitutes an important limitation. However, it should be highlighted that Ioannou et al. (2009) did not observe any significant changes in the arterial compliance, resistance and Zc between the acute and long-term stages in their experiments.

The remodeling process was simulated based on previous literature on the effect of increased afterload on cardiac function. Of note, we chose to keep the dead volume constant during this manipulation, given that data on V_*d*_ changes during remodeling were lacking. *In vivo*, invasive measurements of the LV pressure-volume loop would be needed in order to include such effects in our future work.

In our cardiac model, we assumed that the normalized activation function is not altered during banding and remodeling, although certain studies have shown a significant variation of the normalized elastance with afterload and introduced correction models (Shishido et al., 2000). This feature will be included in our future computational efforts. Finally, other cardiac contractility models have been proposed in the literature, such as the one developed by Lumens et al. (2009) that includes cavity and sarcomere mechanics. The use of such a model might be relevant for the investigation of the LV remodeling process. This possibility will be explored in future works.

## Conclusion

In this study, we investigated acute and long-term effects of proximal aortic compliance decrease on central hemodynamics by leveraging a computational model of the cardiovascular system. We demonstrated that the main mechanism that drives hypertension acutely after banding is enhancing the forward wave, which becomes even more significant after the heart remodels itself to match the increased afterload. Additionally, we showed that after LV remodeling, the stiffer heart generates a forward wave with a significantly steeper upstroke and an earlier peak, which subsequently alters the central pressure and flow wave shapes. These findings provide valuable insights into the mechanisms of hypertension and provoke us to reconsider our understanding of *Type A* and *Type C* pressure phenotypes, often and erroneously attributed solely to the relative contribution of wave reflections.

## Data Availability Statement

The original contributions presented in the study are included in the article/supplementary material, further inquiries can be directed to the corresponding author/s.

## Author Contributions

SP and NS conceived the study. SP analyzed the data and drafted the manuscript. VB, GR, and DA involved in the interpretation of results. All authors revised the manuscript, approved its final version, and agreed to its publication.

## Conflict of Interest

The authors declare that the research was conducted in the absence of any commercial or financial relationships that could be construed as a potential conflict of interest.

## Publisher’s Note

All claims expressed in this article are solely those of the authors and do not necessarily represent those of their affiliated organizations, or those of the publisher, the editors and the reviewers. Any product that may be evaluated in this article, or claim that may be made by its manufacturer, is not guaranteed or endorsed by the publisher.
